# Preoperative Systemic Inflammation Score Predicts the Prognosis of Patients with Upper Tract Urothelial Carcinoma Undergoing Radical Nephroureterectomy

**DOI:** 10.3390/jcm13030791

**Published:** 2024-01-30

**Authors:** Qihao Wang, Jianjun Ye, Zeyu Chen, Xinyang Liao, Xingyuan Wang, Chichen Zhang, Lei Zheng, Ping Han, Qiang Wei, Yige Bao

**Affiliations:** 1 Department of Urology and Institute of Urology, West China Hospital, Sichuan University, Chengdu 610041, China; wangqihaodr@stu.scu.edu.cn (Q.W.); yejianjun2022@stu.scu.edu.cn (J.Y.); doctorczy@wchscu.edu.cn (Z.C.); liaoxinyang@wchscu.edu.cn (X.L.); wangxingyuan@stu.scu.edu.cn (X.W.); zhangchichen@stu.scu.edu.cn (C.Z.); urozhenglei@stu.scu.edu.cn (L.Z.); hanping@scu.edu.cn (P.H.); 2 West China School of Medicine, Sichuan University, Chengdu 610041, China

**Keywords:** systemic inflammation score, prognosis, upper tract urothelial carcinoma, survival outcome, retrospective cohort study

## Abstract

**Background**: To investigate the prognostic significance of systemic inflammation score (SIS) in upper tract urothelial carcinoma (UTUC) in patients undergoing radical nephroureterectomy (RNU). **Methods**: A total of 313 UTUC patients who underwent RNU at West China Hospital from May 2014 to June 2019 were retrospectively analyzed. The predictive value of SIS for relevant endpoints, including overall survival (OS), cancer-specific survival (CSS), and progression-free survival (PFS), was assessed by Kaplan–Meier curves and the Cox proportional hazards model. **Results**: According to inclusion and exclusion criteria, 218 UTUC patients were ultimately included in this cohort study. Statistical analysis shows that increased SIS was significantly associated with higher TNM stage (*p* = 0.017), lower BMI (*p* = 0.037), absence of hemoglobin (*p* < 0.001), and pathologic necrosis (*p* = 0.007). Kaplan–Meier survival curves clearly visually stratified survival for the three outcomes. After adjusting for tumor grade, the multivariate Cox proportional hazards model results showed that SIS was an independent risk factor for poor OS and CSS (HR = 1.89, 95% CI: 1.11–3.21, *p* = 0.0183, HR = 1.89, 95% CI: 1.07–3.33, *p* = 0.0285) in the advanced group. **Conclusions**: SIS was an independent risk factor for OS and CSS after RNU in patients with high-grade UTUC. It may be a novel and conducive tool for preoperative risk stratification and guiding individualized therapy for high-risk UTUC patients.

## 1. Introduction

Upper tract urothelial carcinoma (UTUC) is uncommon compared to bladder cancer, accounting for only 5–10% of all urothelial cancers. However, approximately two-thirds of UTUC patients have invasive disease at diagnosis, indicating that UTUC is characterized by high recurrence rates in spite of its low morbidity [1]. So far, radical nephroureterectomy (RNU) has become the gold standard for surgical treatment of UTUC. Some scholars believe that the opportunity for prolonged patient survival time lies in the use of perioperative neoadjuvant chemotherapy or immunotherapy [2]. Despite the remarkable understanding of the diagnosis and treatment regimen of UTUC, it is still a threatening tumor with a poor prognosis [3].

Nutritional and inflammatory status have a clear impact on the prognosis of cancer patients. Several promising biomarkers have also been used as prediction factors [4]. To date, some promising serological biomarkers have also been used as predictors in patients with various tumors, such as modified Glasgow Prognostic Score (mGPS), neutrophil-to-lymphocyte ratio (NLR), and lymphocyte-to-monocyte ratio (LMR) [5,6,7]. Plasma albumin levels have also been reported as a clinical predictor of cancer [8]. Although serological examination is a routine preoperative examination in hospitals, and the cost is lower than that of traditional imaging and pathology, there are few reports on the combination analysis of multiple indicators. Systemic inflammation score (SIS), as a novel prognostic score indicator, was first proposed by Y Chang to have an impact on the prognosis of patients with clear cell renal cell carcinoma [9], which further combines systemic nutritional status with inflammatory response factors. Furthermore, SIS was found to be used as an alternative to the modified Glasgow Prognostic Score (mGPS) to improve the prediction of clinical outcomes in patients who underwent curative surgical resection of stage I to IV colorectal cancers (CRCs) in the study of Suzuki Y [5]. SIS has also been testified to predict the prognosis of other cancers, for instance, lung cancer [10,11], colorectal cancer [12,13,14], esophageal squamous cell carcinoma [15,16,17], and gastric cancer [18,19,20,21]. So far, no clinical study has found the correlation and prognostic value of SIS for UTUC, which needs further exploration. The purpose of our study is to investigate the prognostic significance of SIS in upper tract urothelial carcinoma patients who have undergone RNU. If SIS could be used as a predictive model in the future, the outcome of patients can be predicted earlier and more conveniently, so that medical intervention can be implemented earlier to prolong the survival of patients with tumors.

## 2. Materials and Methods

### 2.1. Patients and Data

This study obtained approval from the Ethics Committee on Biomedical Research, West China Hospital of Sichuan University (2021-1209; 20 August 2021). Given the retrospective design and the anonymous and confidential nature of the patient data, this study was carried out with a waiver of informed consent from the local institutional review board. A cohort of 313 UTUC patients who underwent radical nephroureterectomy between May 2014 and June 2019 were initially identified from the West China Hospital database.

In this study, specific criteria were applied to exclude patients with incomplete long-term prognostic information, those lacking preoperative values for albumin, lymphocyte, or monocyte cell counts, individuals with pathologically confirmed non-urothelial carcinoma, and patients who underwent neoadjuvant therapy. Subsequently, 218 patients were selected for further analysis. Clinicopathological data, encompassing age, gender, body mass index, smoking status, hematuria, hemoglobin levels, albumin, lymphocyte and monocyte cell counts, as well as tumor characteristics (including site, size, stage, grade, architecture, necrosis, and multifocality), surgery-related variables (such as lymphovascular invasion (LVI), lymph node metastasis (LNM), surgical margin, bladder cuff management), and concomitant bladder cancer, were retrieved from medical records for comprehensive evaluation.

### 2.2. Pathological Evaluation

The specimens obtained through radical nephroureterectomy were independently evaluated by our expert genitourinary pathologists. Tumor grade was assessed in line with the grading system recommended by the World Health Organization/International Society of Urological Pathology (WHO/ISUP) [22] and tumor stage was evaluated based on the American Joint Committee on Cancer (AJCC) tumor–node–metastasis (TNM) staging system [23], respectively. Conclusive pathological evidence established the occurrence of LNM, while the presence of tumor cells within either lymphatic or blood vessels was defined as LVI.

### 2.3. Patients Grouping

Lymphocyte-to-monocyte ratio (LMR) was calculated from the ratio of the lymphocyte counts and monocyte counts within 1 week before RNU. For LMR, we used the cut-off value of 4.44 set by Y Chang when he studied prognostic factors of renal clear cell carcinoma in 2015 [9]. Patients with both albumin < 4.0 g/dL and LMR < 4.44 were grouped in the high-risk group, as reported in the previous studies, and others were in the low-risk group.

### 2.4. Oncological End Points

In our study, we have included overall survival (OS), cancer-specific survival (CSS), and progression-free survival (PFS) as primary endpoints. These endpoints are accompanied by their corresponding events: overall mortality, cancer-specific mortality, and disease progression. OS, CSS, and PFS are defined as the time elapsed from radical nephroureterectomy until death from any cause, death specifically due to cancer, or any disease progression, respectively.

### 2.5. Follow-Up

The follow-up protocol implemented in this study aligns with the recommendations provided by the European Association of Urology. Follow-up interval begins with a three-month interval in the first year after RNU, followed by a six-month interval in the second year. For the remainder of the time, annual follow-up is recommended in the absence of recurrence or atypical symptoms. The routine follow-up assessments include a physical examination, blood and urine laboratory tests, contrast-enhanced computed tomography scans of the chest and abdomen, and cystoscopy.

### 2.6. Statistical Analysis

This analysis employed R 4.2.3 (http://www.R-project.org; accessed on 1 October 2023, The R Foundation, Boston, MA, USA) and EmpowerStats 4.0 (http://www.empowerstats.com; accessed on 1 October 2023, X&Y Solutions, Inc., Boston, MA, USA) statistical packages to analyze the dataset. Continuous variables were described based on medians and interquartile range (IQR), and categorical variables were described based on number and percentage. Student’s *t* test or the Mann–Whitney U test and the chi-square or Fisher’s exact test were utilized to identify significant differences in clinicopathological characteristics. OS, CSS, and PFS were calculated adopting the Kaplan–Meier method and compared using the log-rank test. Additional subgroup analyses were also conducted according to pathological tumor grade. Cox proportional hazard models were used to investigate the associations between SIS and survival outcomes by HRs (95% CI). The results of this study are considered to be statistically significant with a two-sided *p* < 0.05.

## 3. Results

### 3.1. Baseline Characteristics of Total and Grouped Patients

According to the previously established inclusion and exclusion criteria, 218 UTUC patients were included in this cohort study, with a male-to-female ratio of 115:104 and a median age of 69 (range: 61–75) years. In addition, the distribution of tumor grade was 159 (73.61%) cases in clinical high grade and 57 (26.39%) cases in low grade.

Then, 76 patients with both albumin < 4.0 g/dL and LMR < 4.44 were divided into high-SIS groups, and the remaining 142 patients were assigned to low-SIS (*n* = 142). The results of the statistical analysis show that, compared with other patient characteristics, increased SIS was significantly associated with TNM stage (*p* = 0.017), BMI (*p* = 0.037), and pathological findings such as the absence of hemoglobin (*p* < 0.001) and pathologic necrosis (*p* = 0.007).

Detailed clinicopathologic and laboratory characteristics are given in Table 1.

### 3.2. Kaplan–Meier Survival Curves of Three Survival Outcomes

**Figure 1:**
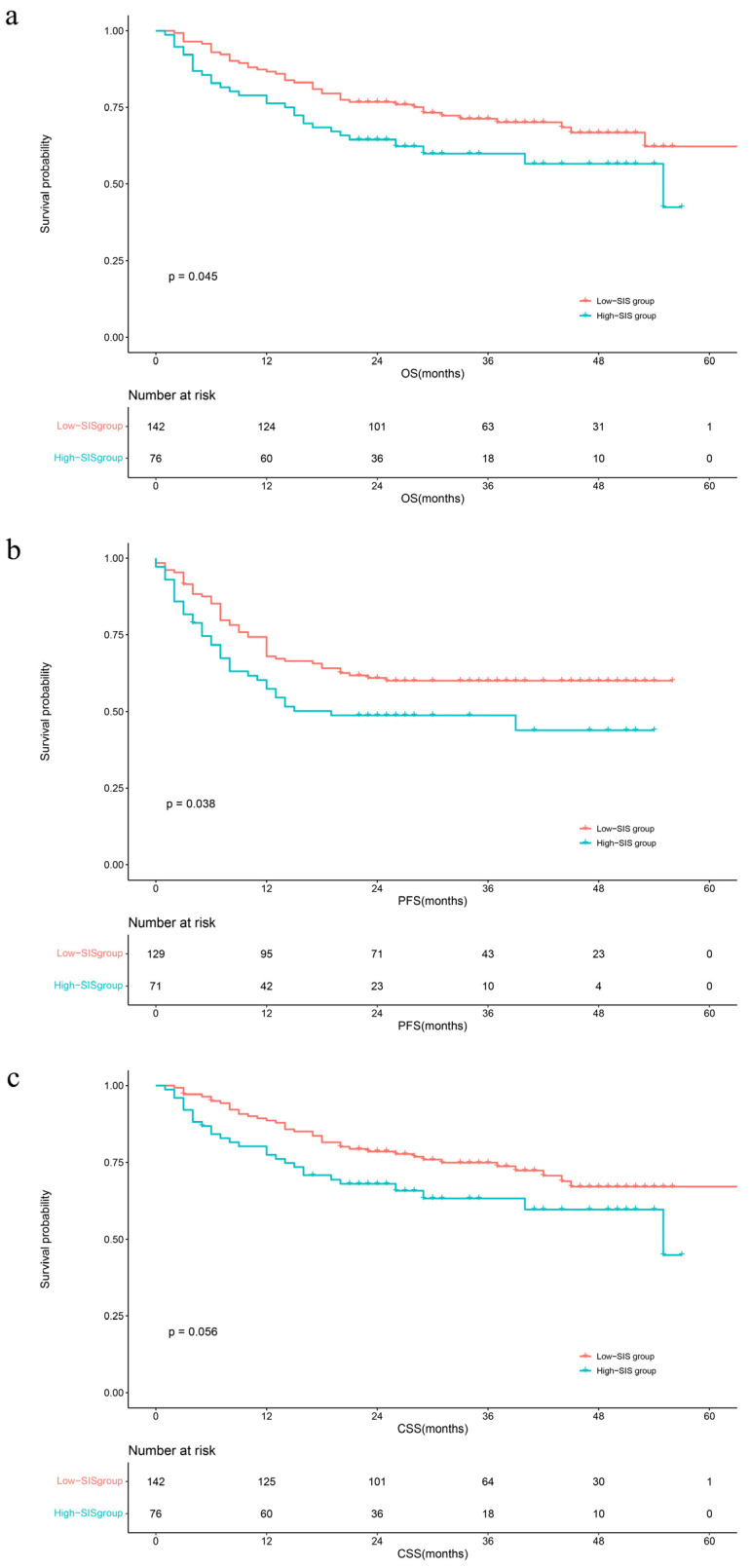
Kaplan–Meier analyses for OS (**a**), PFS (**b**), and CSS (**c**) in UTUC patients according to SIS. Abbreviations: OS = overall survival; PFS = progression-free survival; CSS = cancer-specific survival; SIS = systemic inflammation score.

**Figure 2:**
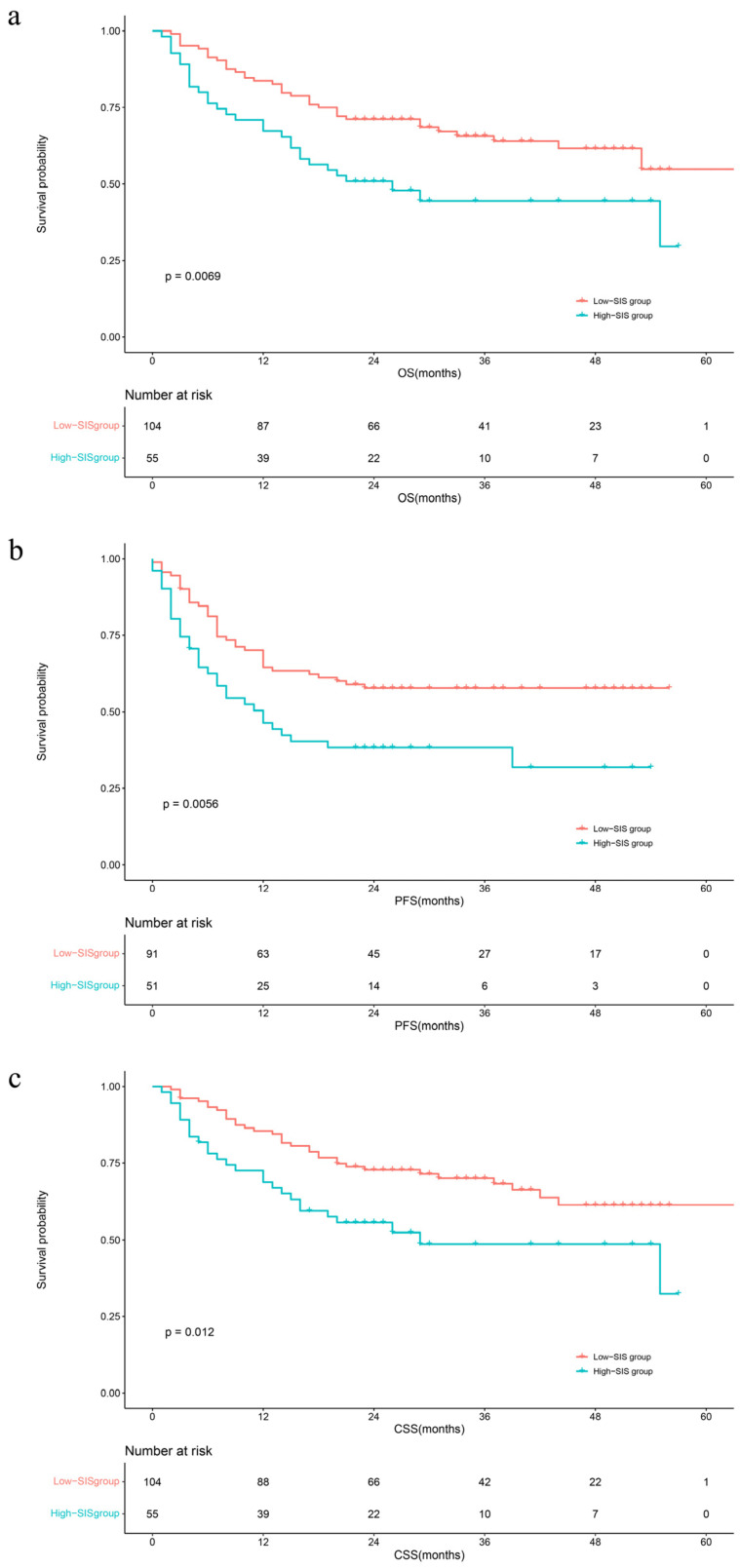
Kaplan–Meier analyses for OS (**a**), PFS (**b**), and CSS (**c**) in high-grade UTUC patients according to SIS. Abbreviations: OS = overall survival; PFS = progression-free survival; CSS = cancer-specific survival; SIS = systemic inflammation score.

The median follow-up time was 29 months (IQR: 20–40). During this period, 75 (34.3%) patients had died from all causes, 68 (31.1%) patients had died from UTUC, and 88 (40.4%) patients had developed disease progression, respectively. The Kaplan–Meier survival curves clearly visually stratify the survival outcomes (OS (log-rank test: *p* = 0.045), CSS (log-rank test: *p* = 0.056) and PFS (log-rank test: *p* = 0.038)) of the UTUC patients with different preoperative SIS scores (Figure 1). The concealed nature of UTUC development determines that most of the patients are already in high grade when they are diagnosed. Therefore, we adjusted the pathological tumor grade to explore the effect of SIS on survival outcomes. The Kaplan–Meier analysis images visually showed greater differences in survival outcomes for subjects with superior SIS scores (Figure 2). Log-rank test results also prove that this discrepancy is meaningful (OS: *p* = 0.0069; CSS: *p* = 0.012; PFS: *p* = 0.0056).

### 3.3. Univariate and Multivariate Cox Regression on Oncological Survival Outcomes

To further elucidate the influencing factors of survival outcomes in UTUC, we analyzed the prognostic value of SIS for OS, CSS, and PFS using Cox regression, and the results are shown in Table 2. We selected the variables (*p* < 0.1) in the univariate analysis and other variables that we were equally interested in for the multivariate Cox regression (Supplementary Table S2). The results of the multivariate analysis showed no significant correlation (all *p* > 0.05) between SIS and three outcomes (OS, CSS, and PFS) (Table 2, Model 1). To further explore the prognostic impact of SIS, the Cox proportional hazards model was also used for the subgroup analysis of the pathological tumor grade. The results showed that SIS was an independent risk factor for poor OS and CSS after the exclusion of other interfering factors in the advanced grade group (HR = 1.89, 95% CI: 1.11–3.21, *p* = 0.018; HR = 1.89, 95% CI: 1.07–3.33, *p* > 0.029) (Table 2, Model 3). The univariate Cox analysis revealed that high SIS was significantly associated with a poorer PFS (HR = 1.90, 95% CI: 1.19–3.05, *p* = 0.008), but this association was not seen in the multivariate analysis (*p* = 0.113). The results of the univariate and multivariate Cox analyses of the remaining clinical characteristics are shown in Supplementary Table S1.

## 4. Discussion

We investigated the laboratory findings of hematologic markers of systemic inflammatory response and prognostic factors of clinicopathological features in 218 patients with upper urothelial carcinoma and found that high-risk SIS was significantly associated with worse physical conditions and tumor characteristics. Moreover, multivariate analysis of the high tumor grade subgroups showed that the subjects with a higher SIS score had significantly poorer overall survival and cancer-specific survival, indicating that inflammation-based scores can also provide important prognoses independent of tumor grade. Therefore, our study demonstrates that the systemic inflammation factors might serve as a novel prognostic factor, thus making the traditional clinical prediction model more comprehensive.

The hypothesis of the relationship between cancer and inflammation was proposed in the 19th century introducing that inflammation has important effects in the prevention and treatment of cancer [24]. In many types of cancer, carcinogenic alterations induce an inflammatory microenvironment that in turn promotes tumor development [25]. Increasing evidence suggests that systemic inflammation mediated by local immune responses plays a role in the prediction of tumor progression and survival outcomes in cancer patients [26]. The systemic inflammation score consists of three laboratory serological markers, including albumin levels, monocyte counts, and lymphocyte counts. Hypoalbuminemia has been shown to be an inferior prognosis indicator for multiple cancers, suggesting not only dystrophic or even cachectic status, but also an increased risk of infections and other diseases [8,27]. Some tumors upregulate inflammatory mediators and recruit other immune cells with tumor-promoting properties, such as monocytes, neutrophils, or innate lymphoid cells (ILCs) [28]. Monocytes are recruited to tumor tissues and differentiate into tumor-associated macrophages (TAMs). TAMs produce a series of cytokines with tumor-promoting activity, which trigger clinical tumor progression by blocking macrophage recruitment or repolarization [29,30]. Moreover, monocytes can also induce tumor angiogenesis, reduce responsiveness to inflammatory stimuli, and suppress antitumor immunity [31]. Lymphocytes prevent the spread and metastasis of tumor cells by establishing the mechanism of tumor suppression, such as senescence surveillance, cancer immune surveillance, and cancer immune editing [32]. The above explains the decreased LMR in a variety of malignant tumors with poor prognosis. Our study showed that high SIS was significantly associated with poor physical condition, and more dangerous tumor features in patients with aggressive tumors, revealing some complicated links between tumor progression and elevated systemic inflammatory response indicators. Accordingly, we consider that high-risk SIS reflects a persistent inflammation state and impaired nutrition, explaining the negative impact on UTUC prognosis.

Traditionally, the prediction of outcome in UTUC patients is based on clinicopathological factors such as tumor grade, TNM stage, or ALDH 1 and SOX 2 of the resection tissue [3]. Current studies indicate that multiple neotype inflammation-related biomarkers can provide additional prognosis for the traditional clinicopathological features of UTUC. Raised preoperative SII (systemic immune inflammation index) has been shown to be [33,34,35,36] independently associated with OS, CSS, RFS, and MFS in UTUC. Other combinations of biomarkers such as PNI (prognostic nutritional index), PLR (platelet-to-lymphocyte ratio), MLR (monocyte-to-lymphocyte ratio) were similarly found to independently predict OS, CSS, RFS, PFS, MFS (metastasis-free survival), and DSS (disease-specific survival) of UTUC patients [33,34,35,37,38,39]. Moreover, a meta-analysis published in 2018 stated that the neutrophil-to-lymphocyte ratio (NLR) was associated with poor RFS, OS, and CSS in patients receiving RNU for UTUC [40]. The characteristics of UTUC determine that the majority of patients are in the high grade, but so far, no study has deeply investigated the prognosis in patients with high-grade UTUC. To the best of our knowledge, our trial is the first one to evaluate the prognosis of the SIS in patients with UTUC. SIS might not be suitable for predicting survival outcomes in the whole population of UTUC, but it can be used as an independent prognostic factor for high-grade UTUC. A significant relationship between SIS and OS, CSS, and DFS was not observed, probably because the properties of UTUC caused most patients receiving RNU to have moderate-advanced/high-grade UTUC at diagnosis [1]. The progression of cancer activates the body’s immune response and leads to changes in blood parameters, while the immune cells in the blood are not yet significantly affected by the tumors for patients with low-grade UTUC. In addition, chemistry therapy may lead to changes in blood cell parameters, so patients receiving neoadjuvant chemotherapy were not included in this study. Our results showed that the odds of poorer OS (*p* < 0.05) of high-SIS patients were 1.89 times higher than patients with low SIS, indicating that SIS can be seen as an independent predictor of upper urothelial carcinoma in the high-grade UTUC population, and then guide medical staff to provide nutritional support and symptomatic treatment program. We also hope that more large samples and prospective studies will prove this conjecture in the future.

In addition to these inflammatory markers mentioned above, we also found some other novel ready-to-use markers, such as CONUT score, fibrinogen, and De Ritis ratio, etc. [41,42,43,44]. The CONUT score contains three modules: serum albumin concentration, lymphocyte count, and total cholesterol concentration, which reflect the nutritional status and the degree of tumor-derived chronic inflammation. It has now been shown to be associated with postoperative prognosis in bladder cancer and upper urothelial carcinoma. In the advanced stage of many tumors, hypoxia occurs, leading to tumor treatment resistance. Plasma fibrinogen, as a novel marker of hypoxia, has been shown to be associated with the prognosis of upper urothelial carcinoma in recent years. In addition, a meta-analysis conducted in 2020 pooled the effect values of several other preoperative serological biomarkers, demonstrating the effectiveness of different types of plasma markers in evaluating UTUC outcomes. The prognostic index SIS, as a novel predictor of clinical outcomes in recent years, combines serum albumin level and monocyte-to-lymphocyte ratio and also evaluates the nutritional and inflammatory status of the body. It is difficult to assess the merits of SIS versus COUNT, but it is certain that the future potential of SIS is significant compared with other one-way scoring systems.

SIS as a novel prognostic evaluation index has recently been found to be associated with postoperative or non-postoperative survival outcomes in multiple tumors. Initially, Chang developed the SIS system to assess the prognosis of clear cell renal cell carcinoma patients undergoing nephrectomy [9]. For non-small-cell lung cancers (NSCLCs) patients undergoing video-assisted thoracoscopic surgery (VATS) lobectomy, multivariate analysis showed that a high SIS score was an unfavorable prognostic factor for OS and DFS [10,11]. Simultaneously, SIS is also an independent prognostic factor for overall survival in colorectal cancer (CRC) patients [5,12]. For rectal cancer patients undergoing adjuvant chemoradiotherapy after total mesorectal excision (TME), marginal statistically significant differences (*p* > 0.05) were observed between the SIS-low and SIS-high groups regarding leukopenia during adjuvant chemoradiotherapy [14]. Preoperative SIS has similarly been shown to be independently associated with poorer OS in gastric cancer patients undergoing radical gastrectomy [18,19]. Moreover, survival outcomes in patients suffering from other cancers, for instance, esophageal cancer, nasopharyngeal carcinoma, hepatocellular carcinoma, cervical cancer, temporal bone squamous cell carcinoma, oral cavity squamous cell carcinoma, breast cancer, and pancreatic ductal adenocarcinoma had already been shown to be independently associated with SIS [45,46,47,48,49,50,51].

Most of the existing articles have proved that increased preoperative SIS is related to adverse clinical outcomes and postoperative complications, but few studies have addressed the relationship between postoperative SIS level and patients’ prognosis. In the study of Lin, preoperative SIS and postoperative SIS were designated as low- and higher-level groups (four groups in total), respectively, and compared. Different conversion forms were divided into three risk levels and concluded that medium and high risk are more prone to adverse OS, local recurrence, and distant metastasis compared with low risk [20]. Another comparative study of SIS after radical resection in patients with II/III phase gastric cancer also confirmed that surgical stress and other postoperative events cause altered hematological parameters, resulting in a greater impact of postoperative SIS on patient survival outcome than preoperative SIS [21]. However, the time of SIS measurement in the two studies was inconsistent, and the hematological parameters in different postoperative stages were also quite different due to the occurrence of perioperative stress and postoperative adverse events. Nowadays, the majority of hospitals have carried out postoperative reviews of periodic blood routine results, which facilitates our perioperative risk stratification and nutritional intervention according to specific serological indicators in the future. It is hoped that future studies with larger samples could reevaluate the differential effects of preoperative SIS and postoperative SIS and SIS levels in different postoperative periods in UTUC and use external data to verify the above views.

Compared with other clinicopathological factors, SIS has the following advantages. Chiefly, SIS is based on the standard parameters of serum albumin, lymphocyte, and monocyte counts, which can be obtained by routine laboratory tests of preoperative and postoperative, so as to reflect the nutritional and systemic inflammatory response in a timelier manner and develop the optimum treatment strategy for the patients. Moreover, SIS can provide an additional prognosis for traditional clinicopathological features, which contribute to better stratifying patients requiring postoperative follow-up or participation in clinical trials. However, limited data exists on the survival benefits of improving albumin, reducing lymphocytes, or improving monocytes in cancer patients.

This study also has some limitations. First, this study is a retrospective, single-center study, and bias is inevitable during the experimental design. Thus, the authors screened the study population strictly according to the previously established inclusion and exclusion criteria and collected all suspected and confirmed data to improve the accuracy and objectivity of the data under the premise of ensuring that the loss rate did not exceed 10%. Future prospective studies in other populations and larger cohorts are needed. Second, the cut-off values of the SIS composition parameters differ between this study and others, which should be further analyzed in later studies. Third, SIS was not horizontally compared with other hematological parameters in this study. It is hoped that more convenient and effective prognostic scoring items can be developed and integrated into daily clinical applications. Fourth, we did not evaluate the association of SIS with preoperative plasma fibrinogen levels and perioperative complications, which might be confounding factors. Fifth, recent studies have shown that the presence of variant histologies is associated with worse survival outcomes for urothelial carcinoma [52,53]. The proportion of data on variant histology in the population included in this study is low, and researchers are currently supplementing these data. In future studies, UTUC variant histology data will be presented and the impact on prognosis will be further analyzed.

## 5. Conclusions

SIS was an independent risk factor for OS after RNU in patients with high-grade UTUC. It may be a novel and conducive tool for preoperative risk stratification and guiding individualized therapy for high-risk UTUC patients.

## Figures and Tables

**Table 1:** Baseline characteristics of included patients.

Characteristics	Total Patients (*n* = 218)	Low-SIS Group (*n* = 142)	High-SIS Group (*n* = 76)	*p*-Value
Age, median (IQR), (years)	69.00 (61.50–75.00)	68.00 (59.25–75.00)	70.00 (64.75–75.25)	0.107
Albumin, median (IQR), (g/L)	40.90 (36.65–43.27)	42.45 (41.00–44.30)	35.10 (32.15–38.10)	<0.001
L/M, median (IQR)	3.46 (2.51–4.87)	4.36 (2.95–5.55)	2.68 (1.49–3.49)	<0.001
BMI, median (IQR) (kg/m^2^)	22.66 (20.73–25.25)	22.99 (21.36–25.33)	21.77 (19.85–24.68)	0.037
Gender, *n* (%)				0.721
Female	104 (47.49%)	69 (48.59%)	35 (46.05%)	
Male	115 (52.51%)	73 (51.41%)	41 (53.95%)	
Smoking status, *n* (%)				0.666
Yes	156 (71.89%)	100 (70.92%)	56 (73.68%)	
No	61 (28.11%)	41 (29.08%)	20 (26.32%)	
Tumor stage, *n* (%)				0.017
Tis/Ta/T1/T2	118 (54.38%)	85 (60.28%)	33 (43.42%)	
T3/T4	99 (45.62%)	56 (39.72%)	43 (56.58%)	
Tumor grade, *n* (%)				0.946
High	57 (26.39%)	37 (26.24%)	20 (26.67%)	
Low	159 (73.61%)	104 (73.76%)	55 (73.33%)	
LNM, *n* (%)				0.355
pN0	16 (7.37%)	13 (9.22%)	3 (3.95%)	
pN1/2	15 (6.91%)	10 (7.09%)	5 (6.58%)	
pNx	186 (85.71%)	118 (83.69%)	68 (89.47%)	
LVI, *n* (%)				0.980
Yes	194 (89.40%)	126 (89.36%)	68 (89.47%)	
No	23 (10.60%)	15 (10.64%)	8 (10.53%)	
Tumor site, *n* (%)				0.471
Renal pelvis	89 (40.64%)	54 (38.03%)	34 (44.74%)	
Ureter	124 (56.62%)	83 (58.45%)	41 (53.95%)	
Both	6 (2.74%)	5 (3.52%)	1 (1.32%)	
Size, *n* (%)				0.070
<3	71 (32.87%)	52 (37.14%)	19 (25.00%)	
≥3	145 (67.13%)	88 (62.86%)	57 (75.00%)	
Multifocal, *n* (%)				0.134
Yes	168 (77.06%)	105 (73.94%)	63 (82.89%)	
No	50 (22.94%)	37 (26.06%)	13 (17.11%)	
Necrosis, *n* (%)				0.007
Yes	208 (94.98%)	139 (97.89%)	68 (89.47%)	
No	11 (5.02%)	3 (2.11%)	8 (10.53%)	
Blood type				0.841
A	80 (36.9%)	55 (39.0%)	25 (32.9%)	
B	47 (21.7%)	30 (21.3%)	17 (22.4%)	
AB	19 (8.8%)	12 (8.5%)	7 (9.2%)	
O	71 (32.7%)	44 (31.2%)	27 (35.5%)	
Hematuria, *n* (%)				0.338
Yes	54 (24.77%)	38 (26.95%)	16 (21.05%)	
No	164 (75.23%)	103 (73.05%)	60 (78.95%)	
Surgery margin, *n* (%)				0.334
Positive	201 (93.06%)	132 (94.29%)	69 (90.79%)	
Negative	15 (6.94%)	8 (5.71%)	7 (9.21%)	
Bladder irrigation				0.110
No	49 (41.2%)	26 (35.1%)	23 (51.1%)	
Unilateral	13 (10.9%)	7 (9.5%)	6 (13.3%)	
Bilateral	57 (47.9%)	41 (31.2%)	27 (35.5%)	
Tumor architecture, *n* (%)				0.773
Sessile	145 (68.40%)	96 (69.06%)	49 (67.12%)	
Papillary	67 (31.60%)	43 (30.94%)	24 (32.88%)	
BCM, *n* (%)				
No	185 (90.69%)	118 (90.08%)	67 (91.78%)	0.688
Yes	19 (9.31%)	13 (9.92%)	6 (8.22%)	
Co-bladder cancer, *n* (%)				
No	89 (44.95%)	55 (42.97%)	34 (48.57%)	0.449
Yes	109 (55.05%)	73 (57.03%)	36 (51.43%)	

Abbreviations: IQR = interquartile range, SIS = systemic inflammation score, L/M = lymphocyte–monocyte ratio, LNM = lymph node metastasis, LVI = lymphovascular invasive, BCM = bladder cuff management.

**Table 2:** The results of Cox regression about the effect of SIS on survival outcomes.

**Group**	**Overall Survival**			
	**Univariate**		**Multivariate**	
	**HR (95% CI)**	***p*** **-Value**	**HR (95% CI)**	***p*** **-Value**
Model 1				
SIS (0 vs. 1)	1.60 (1.00, 2.54)	0.048	1.45 (0.84, 2.49)	0.183
Model 2				
SIS (0 vs. 1)	0.43 (0.05, 3.72)	0.446	NA	NA
Model 3				
SIS (0 vs. 1)	1.92 (1.18, 3.11)	0.009	1.89 (1.11, 3.21)	0.018
**Group**	**Cancer-Specific Survival**			
	**Univariate**		**Multivariate**	
	**HR (95% CI)**	***p*** **-Value**	**HR (95% CI)**	***p*** **-Value**
Model 1				
SIS (0 vs. 1)	1.60 (0.98, 2.60)	0.060	1.55 (0.90, 2.67)	0.112
Model 2				
SIS (0 vs. 1)	0.56 (0.06, 5.04)	0.606	NA	NA
Model 3				
SIS (0 vs. 1)	1.90 (1.14, 3.15)	0.014	1.89 (1.07, 3.33)	0.029
**Group**	**Progression-Free Survival**			
	**Univariate**		**Multivariate**	
	**HR (95% CI)**	***p*** **-Value**	**HR (95% CI)**	***p*** **-Value**
Model 1				
SIS(0 vs. 1)	1.55 (1.02, 2.37)	0.042	1.27 (0.79, 2.04)	0.332
Model 2				
SIS(0 vs. 1)	0.83 (0.29,2.37)	0.730	NA	NA
Model 3				
SIS(0 vs. 1)	1.90 (1.19, 3.05)	0.008	1.48 (0.88, 2.51)	0.142

Model 1: non-adjusted, Model 2: adjusted for pathological low tumor grade, Model 3: adjusted for pathological high tumor grade. Abbreviations: HR = hazard ratio, CI = confidence interval, SIS = Systemic inflammation score, NA = not available due to the limited numbers.

## Data Availability

The data that support the findings of this study are available upon request from the corresponding author, upon reasonable request.

## Supplementary Materials

The following supporting information can be downloaded at: https://www.mdpi.com/article/10.3390/jcm13030791/s1, Figure S1: Flow chart of patients enrolled in this study; Figure S2: Kaplan–Meier analyses for OS (a), PFS (b), and CSS (c) in low-grade UTUC patients according to SIS; Table S1: Univariate and multivariate analysis of SIS on survival outcomes in UTUC patients; Table S2: Univariate and multivariate analysis of SIS on survival outcomes in high-grade UTUC subgroup.
