# Supplementary material for: Preoperative Systemic Inflammation Score Predicts the Prognosis of Patients with Upper Tract Urothelial Carcinoma Undergoing Radical Nephroureterectomy
Source: J Clin Med. 2024 Jan 30;13(3):791. doi: 10.3390/jcm13030791 (PMC10856497; doi:10.3390/jcm13030791)
Supplement: Supplementary file 1 [file jcm-13-00791-s001.zip › Table S1.pdf]

**Supplementary Table S1.** Univariate and multivariate analysis of SIS on survival outcomes in UTUC patients.

| Variables | Overall survival | | | | Cancer-Specific Survival | | | | Progression-free survival | | | |
|---|---|---|---|---|---|---|---|---|---|---|---|---|
| | Univariable | | Multivariable | | Univariable | | Multivariable | | Univariable | | Multivariable | |
| | HR (95%CI) | P value | HR (95%CI) | P value | HR (95%CI) | P value | HR (95%CI) | P value | HR (95%CI) | P value | HR (95%CI) | P value |
| Age | 1.00(0.98, 1.03) | 0.6661 | | | 1.00(0.97, 1.02) | 0.8583 | | | 1.00 (0.98, 1.02) | 0.6743 | | |
| Body mass index | 1.04(0.96,1.12) | 0.3598 | | | 1.05 (0.98, 1.14) | 0.1838 | | | 1.03 (0.97, 1.10) | 0.3300 | | |
| Gender | | | | | | | | | | | | |
| Female | Reference | | | | Reference | | | | Reference | | | |
| Male | 0.72(0.45, 1.13) | 0.1555 | | | 1.31 (0.81, 2.12) | 0.2650 | | | 0.78 (0.51, 1.18) | 0.2376 | | |
| Smoking | | | | | | | | | | | | |
| None/Former | Reference | | | | Reference | | | | Reference | | | |
| Current | 0.86(0.51,1.45) | 0.5805 | | | 0.86 (0.49, 1.48) | 0.5801 | | | 0.76 (0.46, 1.24) | 0.2722 | | |
| Tumor stage | | | | | | | | | | | | |
| <T3 | Reference | | Reference | | Reference | | | | Reference | | Reference | |
| ≥T3 | 4.22(2.53,7.03) | <0.0001 | 2.59 (1.34, 5.03) | 0.0049 | 5.01 (2.87, 8.75) | <0.0001 | 2.88 (1.49, 5.57) | 0.0016 | 3.01 (1.95, 4.65) | <0.0001 | 2.57 (1.45, 4.56) | 0.0012 |
| Tumor grade | | | | | | | | | | | | |
| Low | Reference | | Reference | | Reference | | | | Reference | | Reference | |
| High | 5.14(2.23,11.86) | 0.0001 | 2.58 (0.86, 7.75) | 0.0910 | 5.64 (2.26, 14.05) | 0.0002 | 5.08 (1.65, 15.64) | 0.0046 | 2.03 (1.20, 3.46) | 0.0087 | 0.91 (0.46, 1.83) | 0.8011 |
| LNM | | | | | | | | | | | | |
| pN0 | Reference | | Reference | | Reference | | | | Reference | | Reference | |
| pN+ | 2.88(1.06,7.82) | 0.0376 | 2.06 (0.60, 7.09) | 0.2504 | 7.37(1.61, 33.69) | 0.0100 | 5.96 (1.18, 30.03) | 0.0304 | 11.00 (2.43, 49.80) | 0.0019 | 4.26 (0.91, 19.95) | 0.0660 |
| pNx | 0.78(0.33,1.80) | 0.5571 | 1.08 (0.35, 3.30) | 0.8986 | 2.26 (0.55, 9.27) | 0.2575 | 4.33 (0.96, 19.57) | 0.0572 | 3.25 (0.80, 13.22) | 0.1005 | 2.68 (0.64, 11.29) | 0.1796 |
| LVI | | | | | | | | | | | | |
| No | Reference | | Reference | | Reference | | | | Reference | | Reference | |
| Yes | 2.31(1.26,4.21) | 0.0065 | 1.83 (0.91, 3.70) | 0.0917 | 2.64 (1.44, 4.86) | 0.0018 | 1.51 (0.77, 2.98) | 0.2308 | 1.48 (0.79, 2.78) | 0.2257 | 1.51 (0.77, 2.98) 0.2308 | |
| Tumor site | | | | | | | | | | | | |
| Renal pelvis | Reference | | Reference | | Reference | | | | Reference | | | |
| Ureter | 1.16(0.71,1.90) | 0.5424 | 1.21 (0.66, 2.21) | 0.5411 | 1.13 (0.67, 1.89) | 0.6450 | 0.90 (0.50, 1.64) | 0.7414 | 1.06 (0.70, 1.63) | 0.7726 | | |
| Both | 4.83(1.98,11.77) | 0.0005 | 13.54 (4.49, 40.86) | <0.0001 | 5.26 (2.15, 12.91) | 0.0003 | 17.38 (5.89, 51.33) | <0.0001 | 3.03 (0.73, 12.59) | 0.1273 | | |
| Tumor size | | | | | | | | | | | | |
| <3 | Reference | | Reference | | Reference | | | | Reference | | Reference | |
| ≥3 | 1.79(1.04,3.08) | 0.0361 | 1.05 (0.47, 2.36) | 0.9070 | 1.83 (1.03, 3.25) | 0.0404 | 0.97 (0.49, 1.91) | 0.9230 | 1.69 (1.03, 2.77) | 0.0364 | 1.49 (0.79, 2.82) | 0.2203 |
| Multifocal | | | | | | | | | | | | |
| No | Reference | | | | Reference | | | | Reference | | Reference | |
| Yes | 0.67(0.37,1.21) | 0.1827 | | | 0.75 (0.41, 1.38) | 0.3631 | 0.42 (0.23, 0.75) | 0.0035 | 1.01 (0.62, 1.65) | 0.9746 | 1.10 (0.65, 1.86) | 0.7275 |
| Tumor necrosis | | | | | | | | | | | | |
| No | Reference | | | | Reference | | | | Reference | | | |
| Yes | 0.47(0.11,1.91) | 0.2889 | | | 0.51 (0.13, 2.09) | 0.3511 | | | 0.85 (0.31, 2.31) | 0.7473 | | |
| Blood type | | | | | | | | | | | | |
| A | Reference | | | | Reference | | | | Reference | | | |

| | | | | | | | | | | | | |
|---|---|---|---|---|---|---|---|---|---|---|---|---|
| B | 0.67(0.35,1.28) | 0.2275 | | | 0.63 (0.31, 1.27) | 0.1969 | | | 1.14 (0.63, 2.05) | 0.6653 | | |
| AB | 1.57(0.77,3.21) | 0.2190 | | | 1.38 (0.63, 3.05) | 0.4206 | | | 1.25 (0.59, 2.66) | 0.5587 | | |
| O | 0.79(0.45,1.38) | 0.4027 | | | 0.86 (0.49, 1.53) | 0.6168 | | | 1.27 (0.76, 2.12) | 0.3541 | | |
| Hematuria | | | | | | | | | | | | |
| No | Reference | | Reference | | Reference | | | | Reference | | Reference | |
| Yes | 0.46(0.28,0.74) | 0.0015 | 0.48 (0.26, 0.86) | 0.0136 | 0.46 (0.28, 0.76) | 0.0026 | | | 0.67 (0.42, 1.07) | 0.0950 | 0.65 (0.40, 1.07) | 0.0922 |
| Surgery margin | | | | | | | | | | | | |
| Negative | Reference | | Reference | | Reference | | | | Reference | | Reference | |
| Positive | 2.84(1.40,5.73) | 0.0037 | 2.30 (0.99, 5.31) | 0.0515 | 3.18 (1.57, 6.47) | 0.0014 | 1.99 (0.91, 4.34) | 0.0837 | 2.22 (1.11, 4.44) | 0.0237 | 2.16 (0.99, 4.70) | 0.0535 |
| Bladder irrigation | | | | | | | | | | | | |
| No | Reference | | | | Reference | | | | Reference | | | |
| Unilateral | 1.51(0.54,4.25) | 0.4365 | | | 1.63 (0.57, 4.65) | 0.3606 | | | 1.44 (0.47, 4.42) | 0.5221 | | |
| Bilateral | 0.55(0.24,1.25) | 0.1534 | | | 0.46 (0.19, 1.14) | 0.0925 | | | 0.71 (0.32, 1.55) | 0.3851 | | |
| Tumor architecture | | | | | | | | | | | | |
| Sessile | Reference | | Reference | | Reference | | | | Reference | | Reference | |
| Papillary | 0.19(0.09,0.42) | <0.0001 | 0.24 (0.08, 0.75) | 0.0137 | 0.18 (0.08, 0.42) | <0.0001 | 0.43 (0.17, 1.10) | 0.0787 | 0.54 (0.33, 0.89) | 0.0155 | 0.91 (0.47, 1.75) | 0.7735 |
| BCM | | | | | | | | | | | | |
| No | Reference | | | | Reference | | | | Reference | | | |
| Yes | 0.70(0.28, 1.73) | 0.4380 | | | 0.80 (0.32, 1.99) | 0.6274 | | | 1.21 (0.60, 2.41) | 0.5946 | | |
| Co-bladder cancer | | | | | | | | | | | | |
| No | Reference | | | | Reference | | | | Reference | | Reference | |
| Yes | 0.58(0.35, 0.95) | 0.0288 | 0.58 (0.33, 1.03) | 0.0641 | 0.69 (0.41, 1.15) | 0.1550 | | | 0.36 (0.23, 0.57) | <0.0001 | 0.41 (0.25, 0.67) | 0.0004 |
| SIS(0 vs. 1) | | | | | | | | | | | | |
| Low | Reference | | Reference | | Reference | | | | Reference | | Reference | |
| High | 1.60(1.00,2.54) | 0.0481 | 1.45 (0.84, 2.49) | 0.1827 | 1.60 (0.98, 2.60) | 0.0595 | 1.55 (0.90, 2.67) | 0.1122 | 1.55 (1.02, 2.37) | 0.0422 | 1.27 (0.79, 2.04) | 0.3318 |

[a]**Abbreviations:** UTUC = upper tract urothelial carcinoma, HR = hazard ratio, CI = confidence interval, SIS = systemic inflammation score, LNM = lymph node metastasis, LVI = lymphovascular invasion, BCM = bladder cuff management.
