# Supplementary material for: Preoperative Systemic Inflammation Score Predicts the Prognosis of Patients with Upper Tract Urothelial Carcinoma Undergoing Radical Nephroureterectomy
Source: J Clin Med. 2024 Jan 30;13(3):791. doi: 10.3390/jcm13030791 (PMC10856497; doi:10.3390/jcm13030791)
Supplement: Supplementary file 1 [file jcm-13-00791-s001.zip › Table S2.pdf]

Supplementary Table S2. Univariate and multivariate analysis of SIS on survival outcomes in high-grade UTUC subgroup.

Variables	Overall survival				Cancer-Specific Survival				Progression-free survival			
	Univariable		Multivariable		Univariable		Multivariable		Univariable		Multivariable	
	HR (95%CI)	P value	HR (95%CI)	P value	HR (95%CI)	P value	HR (95%CI)	P value	HR (95%CI)	P value	HR (95%CI)	P value
Age	1.00 (0.98, 1.03)	0.7820			1.00 (0.97, 1.02)	0.6895			0.99 (0.97, 1.01)	0.2948		
Body mass index	1.03 (0.96, 1.12)	0.3829			1.06 (0.98, 1.15)	0.1575			1.02 (0.95, 1.09)	0.6186		
Gender												
Female	Reference				Reference				Reference			
Male	0.91 (0.56, 1.48)	0.7101			1.07 (0.65, 1.77)	0.7880			0.70 (0.43, 1.13)	0.1425		
Smoking												
None/Former	Reference				Reference				Reference			
Current	0.97 (0.56, 1.66)	0.9018			0.94 (0.53, 1.67)	0.8381			0.85 (0.49, 1.46)	0.5470		
Tumor stage												
<T3	Reference		Reference		Reference				Reference		Reference	
≥T3	3.33 (1.89, 5.86)	<0.0001	2.39 (1.21, 4.72)	0.0122	3.94 (2.12, 7.32)	<0.0001	3.68 (1.65, 8.19)	0.0014	2.62 (1.56, 4.42)	0.0003	2.45 (1.24, 4.83)	0.0100
LNM												
pN0	Reference		Reference		Reference				Reference		Reference	
pN+	2.84 (1.05, 7.69)	0.0406	2.83 (0.88, 9.12)	0.0816	7.22 (1.58, 33.00)	0.0108	8.09 (1.34, 48.84)	0.0227	10.60 (2.34, 48.00)	0.0022	4.37 (0.93, 20.57)	0.0620
pNx	1.07 (0.46, 2.50)	0.8759	1.51 (0.53, 4.32)	0.4380	3.17 (0.77, 13.02)	0.1103	6.30 (1.19, 33.36)	0.0303	3.92 (0.96, 16.08)	0.0575	2.73 (0.65, 11.58)	0.1719
LVI												
No	Reference				Reference				Reference			
Yes	1.68 (0.92, 3.09)	0.0928	1.34 (0.68, 2.62)	0.3939	1.91 (1.03, 3.54)	0.0394	1.44 (0.72, 2.86)	0.3023	1.21 (0.64, 2.31)	0.5608		
Tumor site												
Renal pelvis	Reference				Reference				Reference			
Ureter	1.22 (0.74, 2.03)	0.4332	0.92 (0.53, 1.61)	0.7825	1.22 (0.71, 2.07)	0.4718	0.83 (0.44, 1.56) 0.5612		1.14 (0.71, 1.83)	0.6009		
Both	6.03 (2.06, 17.66)	0.0010	13.67 (4.09, 45.71)	<0.0001	6.81 (2.31, 20.13)	0.0005	33.99 (8.72, 132.51)	<0.0001	5.13 (0.68, 38.40)	0.1116		
Tumor size												
<3	Reference				Reference				Reference		Reference	
≥3	1.60 (0.91, 2.81)	0.1008			1.67 (0.92, 3.04)	0.0914	1.00 (0.47, 2.14)	0.9971	1.71 (0.97, 3.04)	0.0652	1.65 (0.72, 3.80)	0.2356
Multifocal												
No	Reference				Reference				Reference			
Yes	0.80 (0.44, 1.47)	0.4762			0.90 (0.49, 1.67)	0.7420			1.04 (0.60, 1.80)	0.8862		
Tumor necrosis												
No	Reference				Reference				Reference			
Yes	0.74 (0.18, 3.02)	0.6695			0.84 (0.20, 3.45)	0.8075			2.01 (0.73, 5.53)	0.1746		
Blood type												
A	Reference				Reference				Reference			
B	0.54 (0.27, 1.09)	0.0864			0.50 (0.23, 1.06)	0.0717	0.62 (0.26, 1.48)	0.2827	0.85 (0.45, 1.64)	0.6360		
AB	1.75 (0.82, 3.71)	0.1461			1.48 (0.64, 3.42)	0.3622	1.81 (0.74, 4.46)	0.1949	1.17 (0.50, 2.74)	0.7106		
O	0.74 (0.42, 1.33)	0.3212			0.86 (0.48, 1.55)	0.6163	0.69 (0.36, 1.35)	0.2783	1.02 (0.58, 1.80)	0.9449		

Hematuria												
No	Reference		Reference		Reference				Reference		Reference	
Yes	0.42 (0.25, 0.70)	0.0009	0.47 (0.27, 0.84)	0.0113	0.41 (0.24, 0.70)	0.0010	0.38 (0.20, 0.69)	0.0016	0.57 (0.34, 0.96)	0.0335	0.64 (0.37, 1.12)	0.1157
Surgery margin												
Negative	Reference		Reference		Reference				Reference		Reference	
Positive	2.06 (1.02, 4.18)	0.0450	2.09 (0.97, 4.50)	0.0601	2.28 (1.12, 4.65)	0.0234	1.92 (0.87, 4.24)	0.1086	1.81 (0.90, 3.64)	0.0985	2.14 (0.98, 4.70)	0.0578
Bladder irrigation												
No	Reference				Reference				Reference			
Unilateral	1.29 (0.42, 4.01)	0.6551			1.37 (0.44, 4.30)	0.5895			0.86 (0.19, 3.86)	0.8479		
Bilateral	0.57 (0.23, 1.44)	0.2377			0.43 (0.15, 1.24)	0.1194			0.60 (0.24, 1.53)	0.2873		
Tumor architecture												
Sessile	Reference				Reference				Reference		Reference	
Papillary	0.25 (0.10, 0.63)	0.0033	0.42 (0.16, 1.13)	0.0870	0.22 (0.08, 0.60)	0.0032	0.50 (0.15, 1.65)	0.2554	0.51 (0.26, 1.00)	0.0505	0.99 (0.38, 2.56)	0.9839
BCM												
No	Reference				Reference				Reference		Reference	
Yes	0.79(0.29, 2.17)	0.6430			0.89 (0.32, 2.46)	0.8230			1.66 (0.76, 3.64)	0.2048		
Co-bladder cancer												
No	Reference				Reference				Reference		Reference	
Yes	0.67(0.40, 1.12)	0.1262			0.78 (0.45, 1.33)	0.3522			0.40 (0.24, 0.68)	0.0007	0.44 (0.25, 0.78)	0.0046
SIS(0 vs. 1)												
low	Reference		Reference		Reference				Reference		Reference	
high	1.92 (1.18, 3.11)	0.0085	1.89 (1.11, 3.21)	0.0183	1.90 (1.14, 3.15)	0.0136	1.89 (1.07, 3.33)	0.0285	1.90 (1.19, 3.05)	0.0075	1.48 (0.88, 2.51)	0.1415

[a]**Abbreviations:** UTUC = upper tract urothelial carcinoma, HR = hazard ratio, CI = confidence interval, SIS = systemic inflammation score, LNM = lymph node metastasis, LVI = lymphovascular invasion,BCM = bladder cuff management.
