# Supplementary figures and images for: Preoperative Systemic Inflammation Score Predicts the Prognosis of Patients with Upper Tract Urothelial Carcinoma Undergoing Radical Nephroureterectomy
Source: J Clin Med. 2024 Jan 30;13(3):791. doi: 10.3390/jcm13030791 (PMC10856497; doi:10.3390/jcm13030791)

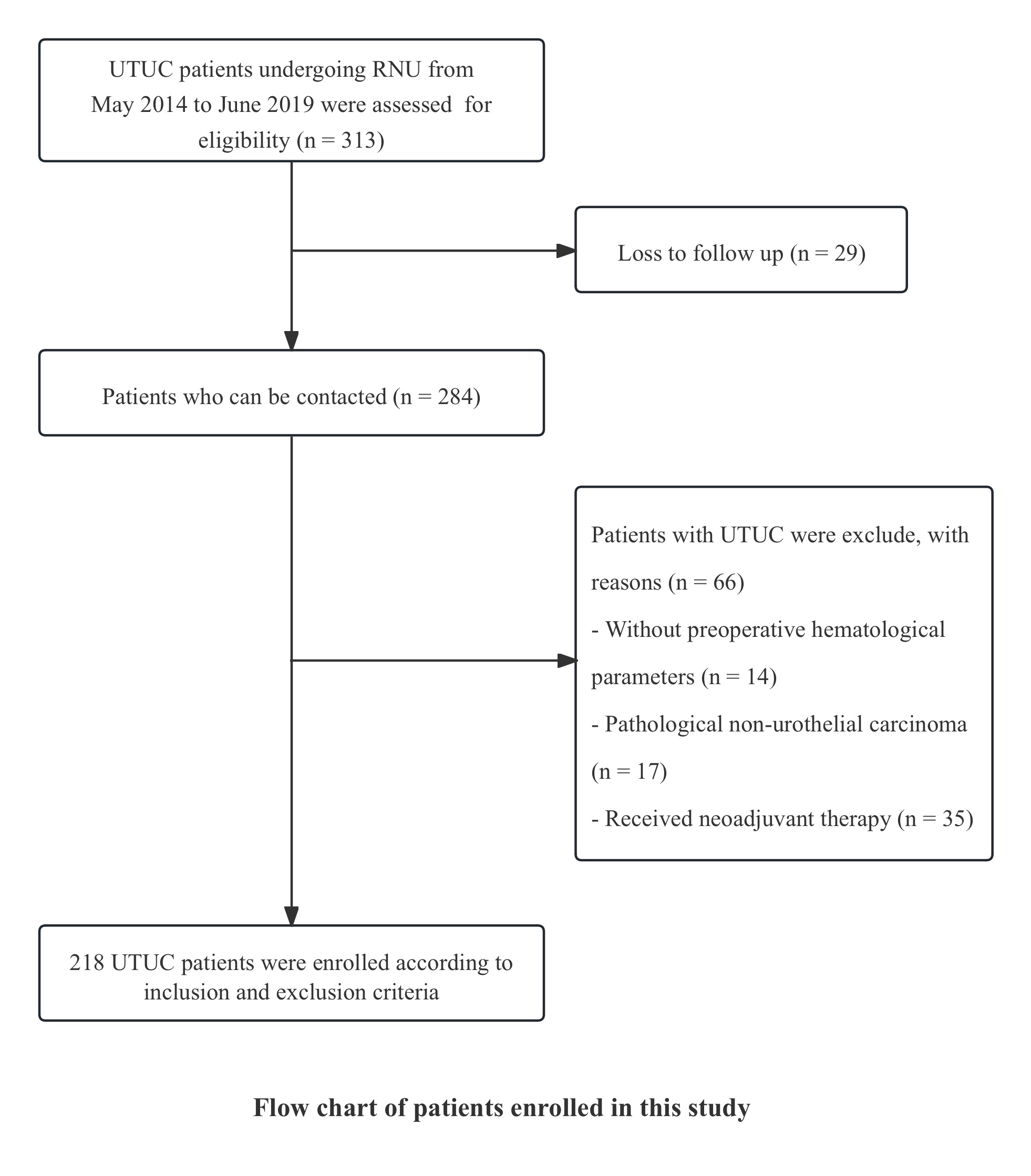

Supplement: Supplementary file 1 [file jcm-13-00791-s001.zip › Figure S1.jpg]

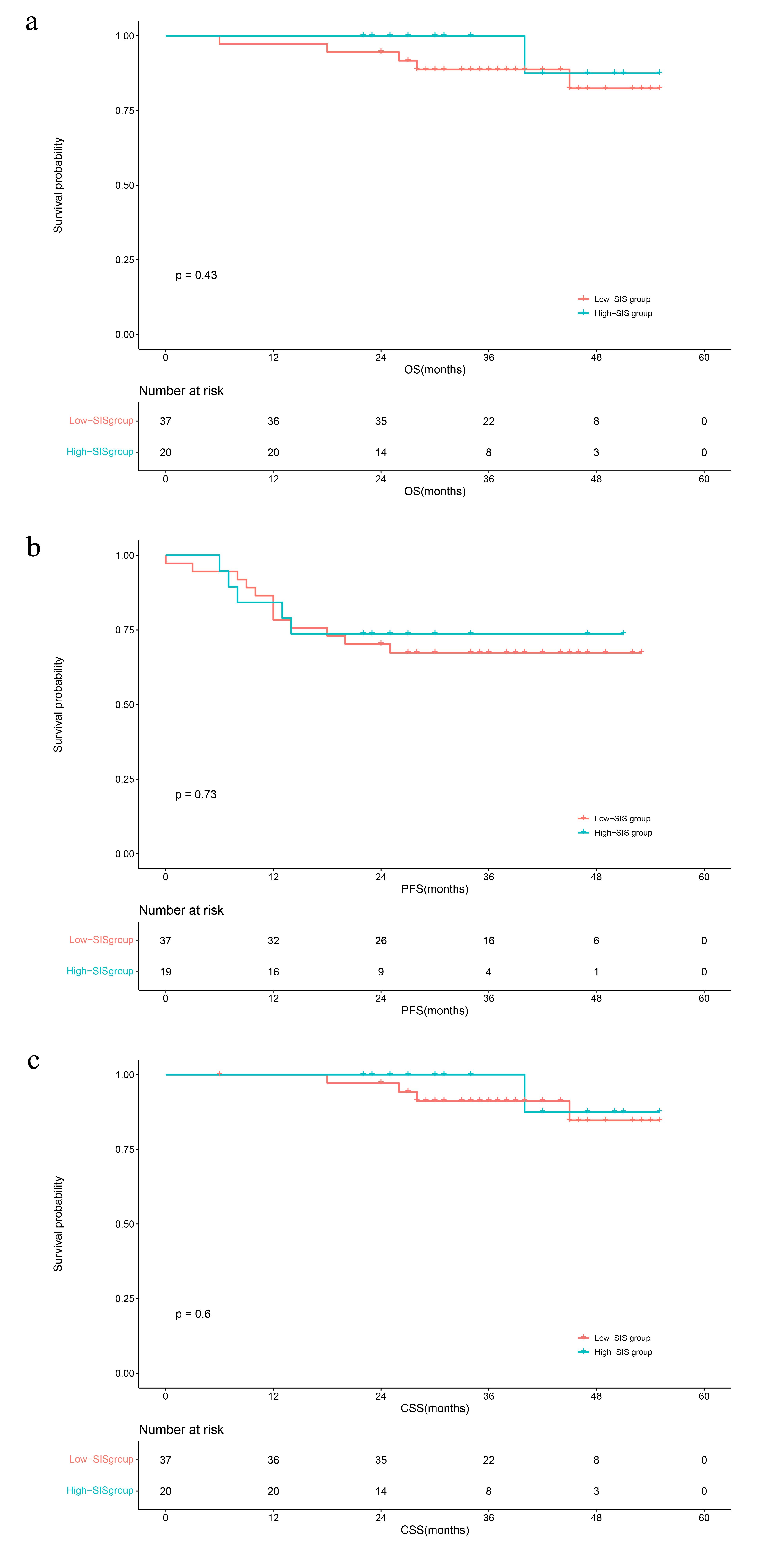

Supplement: Supplementary file 1 [file jcm-13-00791-s001.zip › Figure S2.jpg]
